# Development of a Human IgG1 Monoclonal Antibody Targeting Transferrin Receptor 1 for Antitumor Drug Delivery

**DOI:** 10.3390/antib15020034

**Published:** 2026-04-13

**Authors:** Tingting Ji, Zhaoyun Zong, Ningyuan Gong, Minghui Yan, Shiyu Chen

**Affiliations:** 1 Biotech Drug Research Center, Shanghai Institute of Materia Medica, Chinese Academy of Sciences, Shanghai 201203, China; 2 University of Chinese Academy of Sciences, No. 19A Yuquan Road, Beijing 100049, China; 3 School of Chinese Materia Medica, Nanjing University of Chinese Medicine, Nanjing 210023, China

**Keywords:** antibody phage display, TfR1, antibody-drug conjugate, drug delivery

## Abstract

**Background**: Transferrin receptor protein 1 (TfR1) plays a central role in cellular iron uptake and is frequently overexpressed in malignant tumor cells, rendering it an attractive target for tumor-directed therapy and drug delivery. **Methods**: A fully human single-chain variable fragment (scFv) antibody targeting TfR1, termed T8scFv, was isolated from a human scFv phage display library through three rounds of stringent biopanning and subsequently reformatted into a full-length IgG1 antibody (T8IgG1). Binding kinetics were characterized using Octet biolayer interferometry (BLI), while cellular binding and internalization were assessed by flow cytometry and immunofluorescence microscopy, respectively. T8IgG1 was further conjugated to DT3C, a recombinant truncated diphtheria toxin fusion protein, to evaluate its internalization-dependent cytotoxicity in vitro. **Results**: T8scFv exhibited nanomolar affinity for TfR1 (K_D_ = 214 ± 1 nM), which was substantially enhanced following conversion to the IgG1 format (T8IgG1, K_D_ = 18.5 ± 0.1 nM). T8IgG1 specifically recognized TfR1 on the surface of tumor cells and underwent efficient TfR1-mediated internalization. The T8IgG1-DT3C complex significantly reduced cell viability and induced apoptosis in K562 cells in vitro. **Conclusions**: These findings indicate that T8IgG1 is a moderate-affinity, internalizing anti-TfR1 antibody and highlight its potential as a promising candidate for TfR1-based targeted antitumor drug delivery systems.

## 1. Introduction

**Figure 1:**
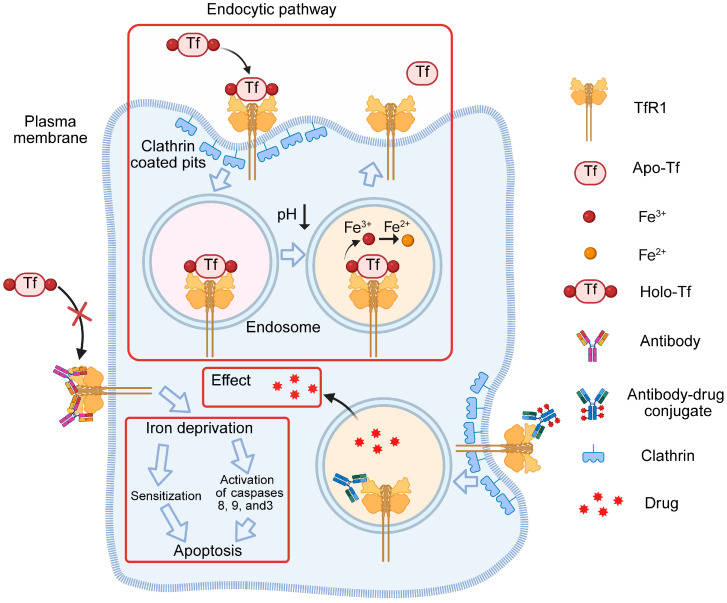
Mechanism of Tf function and TfR1-targeted therapeutic strategies for cancer treatment. Endocytosis of the holo-Tf/TfR1 complex occurs via clathrin-coated pits and the complex is delivered into endosomes. The decrease in endosomal pH (endosomal acidification) induces conformational changes in Tf and TfR1, thereby promoting the release of iron. Apo-Tf remains bound to TfR1 while in the endosome and is released once the complex reaches the cell surface. Antibody-mediated therapeutic strategies targeting TfR1 can be broadly classified into two categories. The first involves an antibody that inhibits the interaction between Tf and TfR1, thereby inducing apoptosis through iron deprivation. The second strategy employs ADC that delivers functional therapeutic payloads via TfR1-mediated internalization, enabling precise and targeted tumor therapy. The arrow labeled “pH” indicates a decrease in acidity, while the other arrows represent subsequent processes; the red boxes are used to distinguish different effect pathways.

Transferrin receptor 1 (TfR1), also known as CD71, is a critical transmembrane glycoprotein involved in the cellular iron uptake pathway [1]. TfR1 is expressed at relatively low levels in most human tissues but is frequently upregulated in a wide range of malignant tumors to meet the increasing iron demands of rapidly proliferating cells; such overexpression is often associated with poor clinical outcomes [2]. Iron, an essential trace element for life, plays indispensable roles in biological processes such as energy metabolism, DNA replication, and cell division. In the circulatory system, iron primarily exists in the form of holo-transferrin (holo-Tf), which binds to TfR1 and is subsequently internalized into cells [3]. The holo-Tf/TfR1 complex undergoes clathrin-mediated endocytosis, one of the most dynamic and active membrane transport processes [2,3,4]. Inside the cell, ions are released and utilized in metabolic pathways, whereas the resulting apo-transferrin (apo-Tf) is recycled back to the bloodstream via receptor-mediated recycling [3] (Figure 1).

TfR1 has been reported to be indispensable for cell survival, as knockout of the TfR1 gene in mice results in embryonic lethality, indicating that there is no compensatory mechanism for iron uptake in the absence of TfR1 [5]. Consequently, the overexpression of TfR1 in tumor cells and their exclusive dependence on TfR1-mediated endocytosis make it a promising target for antibody-conjugate-based therapeutic approaches. Currently, antibody-mediated cancer therapeutic strategies targeting TfR1 can be broadly categorized into two types: (1) blocking the interaction between transferrin (Tf) and TfR1, thereby inducing apoptosis via iron deprivation, and (2) delivering functional therapeutic agents through TfR1-mediated internalization to achieve targeted tumor therapy (Figure 1).

Early TfR1-targeting antibodies, such as the murine/human chimeric antibody ch128.1/IgG1 and its humanized variant hu128.1, have demonstrated significant antitumor activity in xenograft models of multiple myeloma and non-Hodgkin lymphoma, improving overall survival [6,7]. These antibodies exert their effects through inhibition of Tf-TfR1 interactions and potentially Fc-mediated immune mechanisms; however, their clinical efficacy and safety require further validation.

In recent years, TfR1 antibody-conjugate strategies, including antibody–drug conjugates (ADCs), have gained increasing attention, leveraging TfR1-mediated endocytosis to deliver cytotoxic agents directly into tumor cells, thereby enhancing targeted killing while minimizing systemic toxicity. For example, INA03, a humanized anti-CD71 antibody conjugated with Monomethyl Auristatin E, exhibited promising antitumor activity in acute leukemia models but has limitations: its efficacy is highly dependent on high CD71 expression, competitive binding with Tf may interfere with iron metabolism in normal cells, its tissue distribution and cytotoxic payload restrict its applicability, and long-term clinical safety and efficacy remain unverified [8].

Beyond ADCs, TfR1 has also been employed in antibody–oligonucleotide conjugates (AOCs) and brain-penetrating bispecific antibodies (BrainShuttle™ technology), demonstrating potential for nucleic acid delivery and blood–brain barrier (BBB) transport. For instance, TfR1-mediated AOC platforms enable precise delivery of siRNAs or Antisense oligonucleotides (ASOs) to muscle or brain tissues, achieving effective RNA target silencing in mouse and non-human primate models [9,10]; BrainShuttle™ technology fuses a TfR1-binding module with an anti-Aβ antibody, markedly enhancing antibody distribution and efficacy within the brain [11]. These studies indicate the broad applicability of TfR1-mediated delivery systems, though further optimization of antibody affinity, non-competitive binding, and endocytic efficiency is warranted.

In this study, we developed the T8IgG1 antibody, which exhibits several distinct advantages over previous approaches: it binds TfR1 in a non-competitive manner, avoiding disruption of normal Tf-TfR1 interactions and thereby preserving cellular iron metabolism; it has moderate binding affinity (K_D_ = 18.5 ± 0.1 nM) and efficiently undergoes TfR1-mediated internalization for transmembrane delivery; when conjugated to DT3C, it simulates ADC activity in vitro by releasing the DT3C catalytic domain to inhibit protein synthesis and induce tumor cell death, demonstrating superior targeting specificity and delivery efficiency. Compared with previously reported TfR1 antibodies, T8IgG1 combines efficient delivery, low systemic toxicity potential, and non-competitive binding, providing a novel and promising strategy for TfR1-targeted therapy.

## 2. Materials and Methods

### 2.1. Chemical Reagents

DMEM and RPMI 1640 culture media were purchased from Gibco (Grand Island, NY, USA). Fetal bovine serum (FBS), penicillin–streptomycin solution, phosphate-buffered saline (PBS), trypsin without EDTA, and Bis-Tris precast gels were obtained from MeilunBio (Dalian, China). Peptone, yeast extract, and agar powder were purchased from Yeasen (Shanghai, China). The BCA Protein Quantification Kit, bovine serum albumin (BSA), SDS protein lysis buffer, ampicillin sodium, kanamycin, human holo-transferrin, and cell apoptosis–Hoechst staining kit were purchased from Beyotime (Shanghai, China). The 2× MultiF Seamless Assembly Mix was obtained from ABclonal (Wuhan, China). The 2× Phanta Max Master Mix, FastPure EndoFree Plasmid Maxi Kit, and Cell Counting Kit-8 (CCK-8) were purchased from Vazyme (Nanjing, China). TfR1-targeting siRNA and NC siRNA were purchased from GenScript (Nanjing, China). CALNPRNAi in vitro A and CALNPRNAi in vitro B were obtained from Beijing Dona Pharmaceutical Technology (Beijing, China). Ni-NTA beads and rProtein A beads were obtained from Smart-Lifesciences (Changzhou, China). NHS-OG488 was purchased from Shaanxi New Research Bioscience (Xi’an, China).

### 2.2. Cell Culture

K562 cells (were obtained from National Collection of Authenticated Cell Cultures, Chinese Academy of Sciences, Shanghai, China) were cultured in RPMI-1640 medium supplemented with 10% fetal bovine serum and 1% penicillin–streptomycin at 37 °C in a humidified incubator with 5% CO_2_. When the cell confluence reached approximately 80%, the culture medium was replaced, and cells were passaged for subsequent experiments. HepG2 cells (were obtained from National Collection of Authenticated Cell Cultures, Chinese Academy of Sciences, Shanghai, China) were maintained in RPMI-1640 medium containing 10% FBS and 1% penicillin–streptomycin under the same incubation conditions (37 °C, 5% CO_2_, humidified atmosphere). When HepG2 cells reached about 80% confluence, the medium was aspirated, and the cells were washed three times with PBS. The cells were then detached using trypsin and passaged for further experiments.

HEK 293F cells (were obtained from National Collection of Authenticated Cell Cultures, Chinese Academy of Sciences, Shanghai, China) were cultured in OPM-293 CD05 medium at 37 °C with 5% CO_2_ in a shaking incubator. When the cell density exceeded 2 × 10^6^ cells/mL, the culture was diluted and subcultured for subsequent experiments.

### 2.3. Enrichment and Screening of scFv

Biotinylated TfR1 protein was immobilized on streptavidin magnetic beads and used for three rounds of phage display panning with decreasing antigen amounts (10, 5, and 2.5 μg). After blocking, approximately 5 × 10^12^ pfu of the scFv phage library was incubated with the beads for 1 h at room temperature. Unbound phages were removed by extensive PBST washing. Bound phages were eluted with Gly-HCl (pH 2.2), neutralized, and used to infect TG1 cells. Phage output titers were determined by serial dilution plating. The remaining infected bacteria were amplified with M13KO7 helper phage to generate phages for the next round. Input and output titers were recorded for each round.

The scFv phage display library used in this study is a fully human, fully synthetic library that has not undergone immunization, with a theoretical diversity of approximately 2 × 10^9^ variants.

### 2.4. Phage ELISA

Individual monoclonal colonies from the third-round biopanning were inoculated into 96-well plates containing 2YT medium with ampicillin (50 μg/mL) and cultured at 37 °C. Cultures were infected with M13KO7 helper phage and subsequently grown in 2YT medium containing kanamycin and ampicillin (50 μg/mL each) at 30 °C to produce phage supernatants. The supernatants were diluted in 1% BSA-PBST and used for phage ELISA. For ELISA analysis, microtiter plates were coated overnight with TfR1 protein (10 μg/mL), followed by blocking with 5% BSA-PBS prior to incubation with phage-containing supernatants. Bound phages were detected using HRP-conjugated anti-M13 antibody and TMB substrate, and absorbance was measured at 450 nm. Positive clones exhibiting higher OD_450_ values were further cultured, plasmids were extracted, and sequencing was performed to identify scFv antibodies specifically recognizing TfR1.

### 2.5. Expression and Purification of scFv

The scFv coding sequence was subcloned from the phagemid into the pRSF-Duet-1 expression vector, and a C-terminal His_6_ tag was incorporated to facilitate protein purification. To purify the scFv-His_6_ protein, single colonies of BL21 (DE3) harboring the expression plasmid were inoculated into 2YT liquid medium and incubated overnight at 37 °C with shaking at 250 rpm. The overnight culture was diluted 1:100 into 500 mL of fresh 2YT medium containing kanamycin (final concentration 50 µg/mL) and incubated at 37 °C, 220 rpm until the optical density at 600 nm (OD_600_) reached approximately 0.6. The culture was then allowed to stand briefly and rapidly cooled to 30 °C, followed by induction with isopropyl β-D-1-thiogalactopyranoside (IPTG) at a final concentration of 0.5 mM. After shaking at 30 °C, 200 rpm for 12 h, the bacterial cells were harvested. The cell pellet was resuspended in 100 mL of lysis buffer (100 mM Tris-HCl, pH 8.0; 0.7 M sucrose; 0.5 mM EDTA; 10 mg lysozyme) and incubated at 4 °C for 1 h. MgCl_2_ was then added to a final concentration of 5 mM, and the suspension was incubated for an additional 20 min. The lysate was centrifuged at 10,000 rpm for 30 min, and the supernatant was loaded onto a HisTrap HP 1 mL column using an automated sample pump. Weakly bound proteins were washed with 10 mL of buffer A (20 mM Tris-HCl, pH 7.5; 500 mM NaCl; 50 mM imidazole), followed by gradient elution with buffer B (20 mM Tris-HCl, pH 7.5; 500 mM NaCl; 500 mM imidazole). The eluted protein fractions were collected and concentrated to the desired volume using protein ultrafiltration units. To further improve protein purity, the Ni-column-purified target protein was subjected to size-exclusion chromatography using a HiPrep™ Sephacryl™ S-100 HR column (Cytiva, Marlborough, MA, USA) equilibrated with PBS. Protein samples were loaded onto an AKTA Pure chromatography system and separated at a flow rate of 1 mL/min. The elution profile was monitored in real time at 280 nm. Collected elution peaks were analyzed by SDS-PAGE, and protein concentrations were determined using a BCA protein assay kit. Fractions with the highest purity were selected for subsequent experiments.

### 2.6. Construction, Expression, and Purification of the Full-Length IgG1 Format

To enhance the affinity and stability of the antibody against TfR1, the TfR1-specific scFv was engineered into a full-length human IgG1 format, based on the germline sequences of IGHV3-23 and IGKV1-39. GenScript Biotech Corporation (Nanjing, China) synthesized the scFv gene along with the IgG1 heavy-chain and light-chain constant regions, which were assembled into a complete IgG1 gene construct and cloned into an expression plasmid. The plasmid sequence was confirmed by DNA sequencing, and polyethylenimine (PEI) was used to transiently transfect HEK 293F cells cultured in 100 mL of growth medium for full-length antibody expression. After 5 days of culture, the supernatant was collected by centrifugation at 12,000× *g* for 15 min at 4 °C. The collected supernatant was incubated with 500 µL of rProtein A at 4 °C for 12 h, followed by washing with 20 mL of PBS to remove non-specifically bound proteins. Bound antibodies were eluted using 2.5 mL of buffer C (PBS containing 0.1 M glycine, pH 3.0) and immediately neutralized with 280 µL of buffer D (PBS containing 1 M Tris-HCl, pH 8.5). Further purification was performed using HiPrep Sephacryl S-100 HR size-exclusion chromatography (GE Healthcare, Chicago, IL, USA) with PBS as the mobile phase. The target protein-containing fractions were collected and concentrated using an Amicon Ultra-15 centrifugal ultrafiltration device (50 kDa, Merck Millipore, Billerica, MA, USA) at 2300× *g* and 4 °C. Protein concentration was determined using a BCA protein assay kit, and purity was assessed by 10% SDS-PAGE, showing >90% purity. The final protein preparation was stored in PBS buffer.

### 2.7. Determination of Antibody Affinity Constants to TfR1 by Octet BLI

Affinity measurements were performed using the Octet Red 96 system. Biotinylated TfR1 protein (extracellular domain) was diluted in assay buffer (PBS containing 0.02% Tween-20) and immobilized onto streptavidin-coated biosensors until a binding level of approximately 0.6 nm was achieved. Antibodies were serially diluted in assay buffer to cover a concentration range encompassing the expected dissociation constant (K_D_). The biosensors with immobilized target protein were then incubated with antibody solutions at different concentrations for the association phase (120 s), followed by immersion in assay buffer for the dissociation phase (300 s) to monitor binding kinetics in real time. For each interaction, signals were double-referenced: on one hand, streptavidin-coated biosensors immobilized with biotinylated TfR1 were incubated in assay buffer without analyte; on the other hand, streptavidin-coated biosensors without immobilized biotinylated TfR1 were incubated in the presence of analyte. This procedure ensured that the measured RU values reflected only the specific interactions between the target molecules. The association rate constant (k_a_), dissociation rate constant (k_d_), and equilibrium dissociation constant (K_D_) were calculated by globally fitting the experimental data with ForteBio Data Analysis software (version 12.2.2.4).

### 2.8. Protein Conjugation with NHS-OG488

A total of 600 µg of T8IgG1 was incubated with 40 µg of NHS-OG488 (dissolved in 20 µL anhydrous DMSO) at 4 °C in the dark for 3 h to achieve conjugation. Excess NHS-OG488 was removed using a PD-10 desalting column. Eluted fractions containing the target protein were collected, and protein concentration was determined using a BCA protein assay kit. Tf was conjugated with NHS-OG488 using the same procedure. To verify successful conjugation, 1 µg each of T8IgG1-OG488 and Tf-OG488 were analyzed by PAGE.

### 2.9. SiRNA-Mediated Knockdown of TfR1 in K562 Cells

To downregulate TfR1 expression in K562 cells, siRNA-mediated gene silencing was performed. Briefly, 5 × 10^5^ K562 cells were seeded in 6-well plates. Transfection complexes were prepared by mixing 1 μL of TfR1-targeting siRNA (100 μM) with 35 μL of CALNPRNAi in vitro A, followed by the addition of 10 μL of CALNPRNAi in vitro B. The mixture was gently mixed and incubated at room temperature for 5 min to allow complex formation. Subsequently, 154 μL of complete culture medium was added, and the transfection mixture was applied to the cells. Meanwhile cells transfected with a non-targeting control sequence (NC siRNA) served as the control group. After 24 h, cells were centrifuged and the medium was replaced with fresh culture medium. Cells were harvested 48 h post-transfection for subsequent analyses.

### 2.10. Flow Cytometry

Cultured K562 cells were washed twice with wash buffer (PBS containing 0.5% BSA) and resuspended to generate a single-cell suspension. The cell concentration was adjusted to 2 × 10^6^ cells/mL. Fifty microliters of the cell suspension were added to each well of a 96-well plate. Experimental wells received 50 µL of pre-diluted antibody, while control wells received 50 µL of wash buffer. Cells were incubated at room temperature in the dark for 30 min. After incubation, cells were centrifuged at 500× *g* for 6 min at 4 °C, washed twice with wash buffer, and resuspended for analysis. Flow cytometry data were analyzed using FlowJo (version 10.9.0).

### 2.11. Immunofluorescence Assay

To assess antibody internalization, T8IgG1-OG488 was diluted in serum-free DMEM to a final concentration of 10 µg/mL and added to HepG2 cells cultured in 6-well plates until 70–80% confluence (~5 × 10^5^ cells/well) or to K562 cells (~4 × 10^5^ cells/well) in suspension culture. Cells were incubated at 37 °C with 5% CO_2_ for 2 h. After incubation, cells were gently washed three times with pre-chilled PBS to remove uninternalized antibody. Cells were then fixed with 4% paraformaldehyde for 10 min, washed three times with PBS, stained with Hoechst dye for 5 min, washed again, and mounted with mounting medium. Fluorescence images were captured using a fluorescence microscope. Two control groups included cells treated with equivalent amounts of Tf-OG488 or NHS-OG488.

### 2.12. Antibody-Toxin Conjugate Assay

Recombinant DT3C was purified as previously described [12]. The specified amounts of DT3C and purified antibody were pre-incubated in a CO_2_ incubator for at least 30 min. The antibody concentration was fixed at 10 μg/mL across all complex gradients, while DT3C was serially diluted at 0.001, 0.01, 0.1, 1, and 10 μg/mL. The resulting immune complexes were then co-incubated with 5000 K562 cells in a 96-well plate. After 72 h of incubation in a CO_2_ incubator, cell viability was assessed using the CCK-8 assay. Absorbance at 450 nm was measured with a microplate reader, and relative cell viability was calculated. The IC_50_ value of the T8IgG1-DT3C complex was determined by fitting the dose–response curve based on DT3C concentrations.

### 2.13. Competitive ELISA

Competitive ELISA assessment of the potential interference of T8IgG1 with Tf–TfR1 binding. Tf (0.5 μg/well) was immobilized on ELISA plates at 4 °C overnight and subsequently blocked. Biotinylated TfR1 (100 nM) was pre-incubated with varying concentrations of T8IgG1 at 37 °C for 1 h, and the mixtures were then added to the Tf-coated wells for an additional 1 h. Wells incubated with biotinylated TfR1 (100 nM) alone served as the control. After washing, bound biotinylated TfR1 was detected using streptavidin-HRP (1:5000), followed by TMB development and termination with concentrated H_2_SO_4_. Absorbance was measured at 450 nm.

### 2.14. Statistical Analysis and Writing

Statistical analyses were performed using GraphPad Prism (version 8.0.2). Experimental data are presented as mean ± SEM. Two-group comparisons were evaluated using a two-tailed Student’s *t*-test, while multiple group comparisons were analyzed by one-way ANOVA. Differences were considered statistically significant at *p* < 0.05. Portions of the manuscript’s language were reviewed and refined with the assistance of ChatGPT-5. The AI tool was not used in the generation, analysis, or presentation of any scientific data, content, or images.

## 3. Results

### 3.1. Selection of TfR1-Targeting scFv

**Figure 2:**
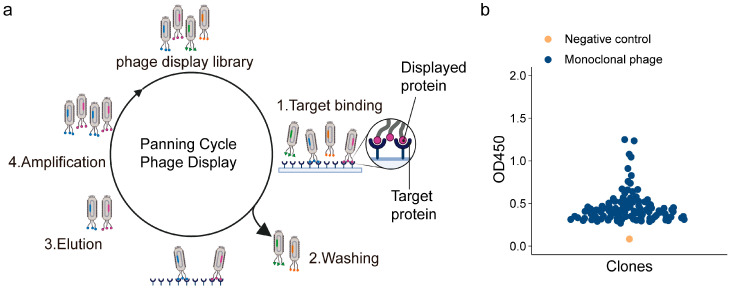
Selection of scFvs targeting TfR1. (**a**) Schematic of the three-round affinity-based biopanning process. (**b**) Phage ELISA results are shown, where blue points denote individual monoclonal phages isolated during screening, and yellow points represent the negative control (1% BSA-PBST). The *y*-axis represents OD_450_ readings.

Using the TfR1 target protein (Figure S1), three rounds of biopanning were performed on the fully human scFv phage display library previously established in our laboratory (Figure 2a).

Monitoring the recovery rate across each round revealed a significant increasing trend, indicating effective enrichment of target-specific phage clones (Table S1). Subsequently, approximately 100 individual clones obtained from the third round were assessed by phage ELISA (Figure 2b), showing that multiple clones exhibited moderately strong target-binding activity. Among these positive clones, we identified the T8scFv, which was further subjected to detailed characterization assays.

### 3.2. Expression of scFv and Full-Length IgG1 Antibodies and Determination of Binding Affinity

**Figure 3:**
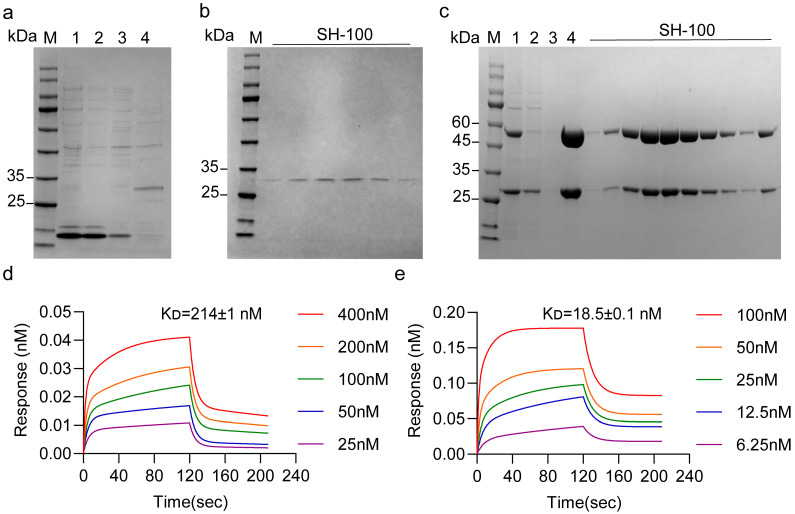
Expression of scFv and Full-Length IgG1 Antibodies and Determination of Binding Affinity. (**a**) SDS-PAGE analysis of T8scFv Ni-NTA–enriched samples under reducing conditions (+DTT), with the target molecular weight of 27 kDa. Lane M, protein marker; Lane 1, whole-cell lysate; Lane 2, flow-through; Lane 3, wash fraction; Lane 4, elution fraction. (**b**) SDS-PAGE analysis of fractions collected from SH-100 size-exclusion chromatography under reducing condition (+DTT). Lane M, protein marker; Other lanes, fractions collected from SH-100 size-exclusion chromatography. (**c**) SDS-PAGE analysis of T8IgG1 rProtein A enriched samples and fractions collected from SH-100 size-exclusion chromatography under reducing condition (+DTT). The target molecular weight of the heavy chain is 50 kDa, and that of the light chain is 25 kDa; the presence of protein glycosylation may cause slight shifts in the observed molecular weights. Lane M, protein marker; Lane 1, culture supernatant; Lane 2, flow-through; Lane 3, wash fraction; Lane 4, elution fraction; Other lanes, fractions collected from SH-100 size-exclusion chromatography. (**d**) Octet BLI measurement of the binding affinity (K_D_) between T8scFv and TfR1. (**e**) Octet BLI measurement of the binding affinity (K_D_) between T8IgG1 and TfR1.

In this study, a periplasmic expression strategy was employed, wherein the PelB signal peptide guided nascent scFv molecules to the more oxidizing periplasmic space, thereby promoting correct folding and disulfide bond formation. This expression system successfully yielded soluble T8scFv protein (Figure 3a,b). Subsequent measurement using BLI demonstrated that the obtained T8scFv bound to TfR1 with an affinity of 214 ± 1 nM (Figure 3d). Detailed fitting parameters are provided in Figure S5 and Table S2.

To further enable in vivo pharmacological functionality, enhance stability, and prolong the half-life of T8scFv to meet the criteria of a therapeutic antibody, the scFv was engineered into a full-length IgG1 format (Figure S2). Following expression in HEK 293F cells, the full-length antibody T8IgG1 was obtained (Figure 3c). The binding affinity of the antibody T8IgG1 to the TfR1 protein was also determined (K_D_ = 18.5 ± 0.1 nM), demonstrating a significant improvement in affinity (Figure 3e).

### 3.3. Flow Cytometric Validation of the Binding Specificity of T8IgG1-OG488 to TfR1

**Figure 4:**
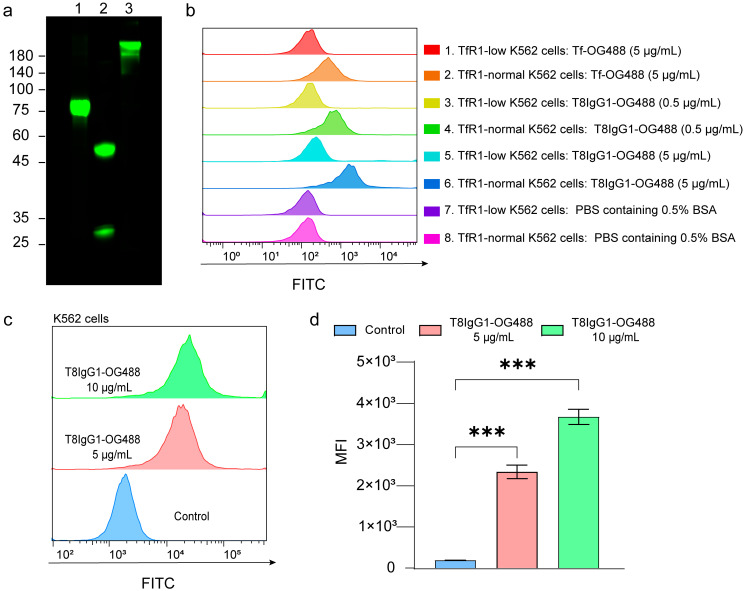
Flow cytometric validation of the binding specificity of T8IgG1-OG488 to TfR1. (**a**) After separation by SDS-PAGE, the proteins were visualized using the FITC channel on a gel imaging system. Lane 1, Tf conjugated with NHS-OG488 under reducing condition (+DTT); Lane 2, T8IgG1 conjugated with NHS-OG488 under reducing condition (+DTT); Lane 3, T8IgG1 conjugated with NHS-OG488 under non-reducing condition (−DTT). (**b**) TfR1 expression in K562 cells was silenced using TfR1-targeting siRNA (TfR1-low K562 cells), while cells transfected with a non-targeting control sequence (NC siRNA) served as the control group (TfR1-normal K562 cells). The experimental conditions were as follows: 1, TfR1-low K562 cells incubated with Tf-OG488 (5 µg/mL); 2, TfR1-normal K562 cells incubated with Tf-OG488 (5 µg/mL); 3, TfR1-low K562 cells incubated with T8IgG1-OG488 (0.5 µg/mL); 4, TfR1-normal K562 cells incubated with T8IgG1-OG488 (0.5 µg/mL); 5, TfR1-low K562 cells incubated with T8IgG1-OG488 (5 µg/mL); 6, TfR1-normal K562 cells incubated with T8IgG1-OG488 (5 µg/mL); 7, TfR1-low K562 cells treated with PBS containing 0.5% BSA; 8, TfR1-normal K562 cells treated with PBS containing 0.5% BSA. (**c**,**d**) Representative flow cytometry images and quantitative analysis of K562 cells. The control group was not labeled with antibody, while the experimental groups were labeled with 5 µg/mL and 10 µg/mL of T8IgG1-OG488, respectively. Experiments were repeated three times, and data are presented as mean ± SEM; *** *p* < 0.001.

To further assess its binding to tumor cells, T8IgG1 was conjugated with OG488 to generate T8IgG1-OG488 (Figure 4a). Flow cytometry analysis demonstrated that T8IgG1-OG488 effectively bound to TfR1 on the surface of K562 cells (Figure 4c), with mean fluorescence intensity significantly higher than that of the negative control group (Figure 4d).

To evaluate the binding specificity of T8IgG1-OG488 to its target, we employed TfR1-targeting siRNA to downregulate TfR1 expression in K562 cells, using cells transfected with a non-specific control sequence (NC siRNA) as the negative control (Figure S6). Following incubation of T8IgG1-OG488 with both groups of cells, fluorescence analysis revealed that the mean fluorescence intensity was markedly reduced in the TfR1-knockdown group compared with the control group (Figure 4b). These results indicate that T8IgG1-OG488 specifically binds to TfR1 expressed on the K562 cell membrane.

### 3.4. Immunofluorescence Analysis of Antibody Internalization

**Figure 5:**
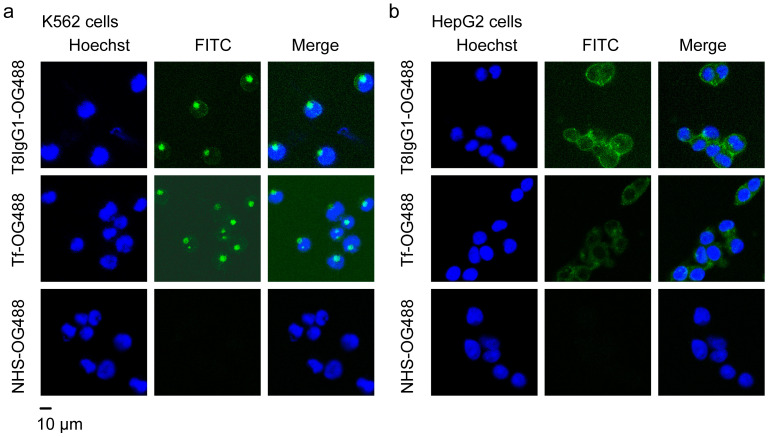
Evaluation of antibody internalization. T8IgG1-OG488 was incubated with K562 (**a**) and HepG2 (**b**) cells for 2 h, followed by PBS washing and cell fixation. Nuclei were stained with Hoechst, and FITC signal distribution was observed under a fluorescence microscope. Green fluorescence indicates the location of T8IgG1-OG488, while blue fluorescence marks the nuclei. The green fluorescence is predominantly distributed in the cytoplasmic region, indicating strong cellular internalization capability of the antibody. In the positive control group with Tf-OG488, green fluorescence is also observed in the cytoplasm. In the negative control group treated with NHS-OG488 alone, no significant green signal is detected, further confirming that the internalization capability is an intrinsic property of T8IgG1.

To evaluate the cellular internalization of the antibodies, K562 and HepG2 cells were incubated with Tf-OG488 and T8IgG1-OG488, respectively, and subsequently prepared for imaging under a confocal microscope. The results showed that in the negative control group, free fluorophore OG488 alone was unable to efficiently enter K562 or HepG2 cells. In contrast, strong green fluorescence was observed within K562 and HepG2 cells treated with Tf-OG488 or T8IgG1-OG488 (Figure 5a,b), with punctate intracellular localization in K562 cells and a more diffuse fluorescence pattern in HepG2 cells, indicating that both Tf-OG488 and T8IgG1-OG488 were effectively internalized by these cells. This suggests their potential capability for intracellular drug delivery.

### 3.5. K562 Cells Exhibit High Sensitivity to the T8IgG1-DT3C Complex

DT3C is a recombinant protein composed of the diphtheria toxin (DT) lacking its receptor-binding domain and the C1, C2, and C3 domains of streptococcal protein G (3C). The DT3C-antibody immunocomplex is internalized only when the antibody specifically recognizes the target antigen expressed on the cell surface.

**Figure 6:**
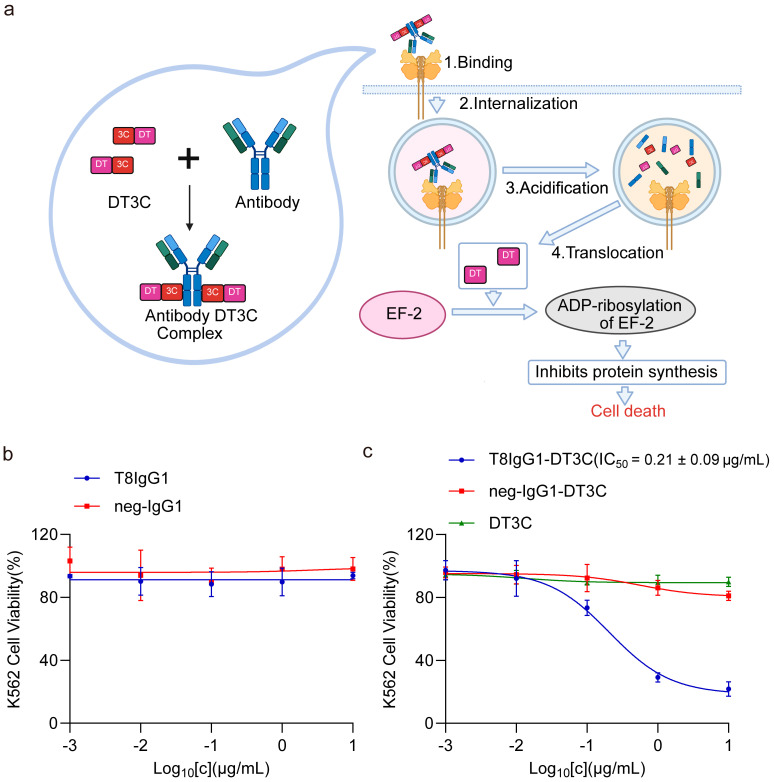
K562 cells exhibit high sensitivity to the T8IgG1-DT3C complex. (**a**) Mechanism of action of the antibody-DT3C complex. (**b**) CCK-8 assay results of 5000 K562 cells treated with T8IgG1 or neg-IgG1 isotype control for 72 h. (**c**) CCK-8 assay results for 5000 K562 cells treated with T8IgG1-DT3C, neg-IgG1-DT3C, or DT3C alone for 72 h.

Upon internalization, DT3C is cleaved by cytosolic furin proteases at its translocation domain, releasing the catalytic domain into the cytoplasm. The catalytic domain of DT3C subsequently ADP-ribosylates elongation factor EF-2, thereby inhibiting protein synthesis and inducing cell death (Figure 6a). Administration of T8IgG1 alone to K562 cells for 72 h did not affect cellular proliferation (Figure 6b). Competitive ELISA results showed that, within the tested concentration range of T8IgG1, there was no significant difference in OD_450_ between the treatment and control groups, indicating that under the experimental conditions, T8IgG1 did not detectably interfere with the binding of Tf to biotinylated TfR1 (Figure S8). This is likely attributable to T8IgG1 does not competitively bind Tf to TfR1; therefore, treatment of K562 cells with T8IgG1 alone does not disrupt iron metabolism or induce cell death. In contrast, treatment with the T8IgG1-DT3C complex for 72 h effectively induced apoptosis in K562 cells (IC_50_ = 0.21 ± 0.09 μg/mL), whereas the control neg-IgG1-DT3C did not affect cell growth or proliferation (Figure 6c).

## 4. Discussion

In this study, a series of three rounds of biopanning and Phage ELISA enabled the effective enrichment and selection of scFv targeting TfR1 (Figure 2a,b). The resulting T8scFv exhibited substantial binding activity in preliminary assays, although its affinity remained moderate (K_D_ = 214 ± 1 nM) (Figure 3d). Following conversion to a full-length IgG1 format, the binding affinity of T8IgG1 markedly increased to 18.5 ± 0.1 nM (Figure 3e). This improvement can be attributed, on one hand, to the critical role of the Fc region in stabilizing antigen binding, and on the other hand, to the fact that the scFv possesses only a single binding site, whereas the IgG1 antibody is bivalent. The binding affinities of antibodies targeting TfR1 reported in the literature cover a wide range, from sub-nanomolar to several hundred nanomolar, indicating significant variability among different TfR1 antibodies [6,7,8,13,14]. The T8IgG1 antibody described in this study belongs to the moderate-affinity category. A key property of an antibody for use in ADC is the efficiency of antibody–antigen complex internalization. Immunofluorescence results demonstrated that T8IgG1 could be internalized by both K562 and HepG2 cells (Figure 5a,b), consistent with the known endocytic mechanism of TfR1. In K562 cells, the fluorescence signal predominantly appeared as punctate intracellular structures, which may reflect accumulation of the antibody–receptor complex within early and late endosomal compartments. This pattern is consistent with the relatively slower recycling kinetics of TfR1 and the tendency toward receptor clustering reported in suspension hematopoietic cells [15,16]. In contrast, HepG2 cells exhibited a more diffuse fluorescence distribution, which may be attributed to faster TfR1 recycling, broader cytoplasmic dispersion, or differences in endocytic trafficking pathways characteristic of adherent hepatic cells [16]. As a constitutively endocytosing receptor, TfR1 has been exploited by multiple antibodies for drug or toxin delivery [17,18,19,20,21,22]. In this study, T8IgG1 did not compete with Tf for TfR1 binding and consequently did not affect cellular proliferation (Figure 6b and Figure S8). However, when conjugated to the toxin DT3C, T8IgG1 effectively delivered DT3C into K562 cells and induced cell death, whereas the control group showed no effect (Figure 6c). These findings demonstrate the targeting specificity and delivery capability of T8IgG1, providing an experimental basis for further development of TfR1-targeted antitumor drug delivery systems. Compared with INA03, which competitively binds Tf to engage TfR1 [8], T8IgG1 does not compete with Tf for TfR1, and therefore does not interfere with cell proliferation. Consequently, it is theoretically associated with lower non-specific toxicity and exhibits superior safety as an anti-tumor drug delivery carrier.

There are several critical limitations in this study. Except for T8scFv, other full-length antibodies derived from scFv sequences (all obtained from the same phage display screening) often exhibit lower affinity, which may result from the flexible linker connecting the VH (heavy chain variable domain) and VL (light chain variable domain) in scFv. In full-length antibodies, the VH and VL interact with the constant regions (CH1/CL), potentially altering the conformation of the complementarity-determining regions (CDRs) and reducing antigen-binding affinity [23,24,25]. While T8IgG1 does not competitively bind Tf, the exact epitope on TfR1 has not been determined; future epitope mapping is necessary to elucidate the antibody-TfR1 interaction mechanism. Additionally, in this study, DT3C was used as the effector molecule for antibody conjugation, linked via the Fc region of the antibody and the 3C region of streptococcal protein G. In practical ADC development, the conjugation strategy can profoundly influence pharmacokinetics, safety, and stability [26]. As a platform for ADC, T8IgG1 warrants exploration of alternative conjugation strategies, such as site-specific conjugation [27,28]. Furthermore, this study was limited to in vitro experiments, and additional preclinical in vitro and in vivo studies are ongoing to evaluate the efficacy and potential toxicity of T8IgG1-based conjugates.

## 5. Conclusions

This study focused on TfR1, a key mediator of iron transport that is highly expressed in various tumor cells, exhibits efficient and stable endocytic properties, and has long been regarded as an ideal molecular target for tumor-targeted therapy and drug delivery.

Using the previously established human scFv phage display library, several candidate clones with favorable binding activity to TfR1 were selected. The T8scFv identified in this study was further expressed in mammalian cells as a full-length IgG1 antibody, T8IgG1, which retained strong target-binding activity, demonstrating the feasibility and practicality of scFv as an initial screening scaffold. Flow cytometry analysis indicated that T8IgG1 specifically recognized TfR1 on tumor cell surfaces. Immunofluorescence microscopy further confirmed that the antibody could be efficiently internalized via TfR1-mediated endocytosis. T8IgG1 does not competitively bind Tf to TfR1; therefore, treatment of K562 cells with T8IgG1 alone does not disrupt iron metabolism or induce cell death. Moreover, T8IgG1 complexed with DT3C exhibited potent cytotoxic effects in K562 cells. Collectively, these results suggest that T8IgG1 holds potential as a delivery vehicle for antitumor drugs and other therapeutic biologics in future studies.

## Data Availability

The original contributions presented in this study are included in the article. Further inquiries can be directed to the corresponding author.

## Supplementary Materials

The following supporting information can be downloaded at: https://www.mdpi.com/article/10.3390/antib15020034/s1, Figure S1: SDS-PAGE analysis of TfR1 purification and biotinylation. Figure S2: Amino acid sequences of T8IgG1 library; Figure S3: The original SDS-PAGE images of T8scFv and T8IgG1, along with the analysis results of the densitometry readings of the target bands. Figure S4: Fluorescent scanning of the original SDS-PAGE of Tf-OG488 and T8IgG1-OG488, along with the analysis results of the densitometry readings of the target bands. Figure S5: Immobilization, association–dissociation, and corresponding fitted kinetic curves of T8scFv and T8IgG1 in Octet experiments. Figure S6: Western blot analysis of TfR1 expression suppression by TfR1-targeting siRNA in K562 cells. Figure S7: Evaluation of antibody internalization with bright-field images. Figure S8: Competitive ELISA assessment of the potential interference of T8IgG1 with Tf–TfR1 binding. Table S1: The phage library titer input and output numbers of each panning. Table S2: Kinetic parameters of T8scFv and T8IgG1 determined by Octet analysis.

## Abbreviations

The following abbreviations are used in this manuscript:

IgG1	Immunoglobulin G1
TfR1	Transferrin receptor protein 1
scFv	Single-chain variable fragment
ADC	Antibody–drug conjugate
AOCs	Antibody–oligonucleotide conjugates
BBB	Blood–brain barrier
ASOs	Antisense oligonucleotides
holo-Tf	Holo-transferrin
apo-Tf	Apo-transferrin
Tf	Transferrin
FBS	Fetal bovine serum
PBS	Phosphate-buffered saline
BSA	Bovine serum albumin
CCK-8	Cell Counting Kit-8
IPTG	Isopropyl β-D-1-thiogalactopyranoside
PEI	Polyethylenimine
DT	Diphtheria Toxin
3C	The C1, C2, and C3 domains of streptococcal protein G
VH	Heavy chain variable domain
VL	Light chain variable domain
CH1/CL	Constant regions
CDRs	Complementarity-determining regions
