# Supplementary material for: Development of a Human IgG1 Monoclonal Antibody Targeting Transferrin Receptor 1 for Antitumor Drug Delivery
Source: Antibodies (Basel). 2026 Apr 13;15(2):34. doi: 10.3390/antib15020034 (PMC13114112; doi:10.3390/antib15020034)
Supplement: Supplementary file 1 [file antibodies-15-00034-s001.zip › antibodies-4211162-supplementary.pdf]

Supporting information

Development of a human IgG1 monoclonal antibody targeting transferrin receptor 1 for antitumor drug delivery

Tingting Ji [1, 2], Zhaoyun Zong [1], Ningyuan Gong [3, 1], Minghui Yan [1, 2] and Shiyu Chen [1, 2, *]

[1] Biotech Drug Research Center, Shanghai Institute of Materia Medica, Chinese Academy of Sciences, Shanghai 201203, China

[2] University of Chinese Academy of Sciences, No. 19A Yuquan Road, Beijing 100049, China

[3] School of Chinese Materia Medica, Nanjing University of Chinese Medicine, Nanjing, 210023, China

* Correspondence: chenshiyu@simm.ac.cn;

Phone: +86 (21) 6807 7979

Fax: +86 (21) 5080 6898

E-mail: chenshiyu@simm.ac.cn

Keywords: Antibody phage display; TfR1; antibody-drug conjugate; drug delivery

Round of Screening	Input Number of Phage (pfu)	Output Number of Phage (pfu)	recovery rate [a]
1	5.0×10^{12}	1.8×10^{6}	3.6×10^{-7}
2	4.0×10^{12}	4.0×10^{7}	1.0×10^{-5}
3	8.0×10^{12}	1.2×10^{9}	1.5×10^{-4}

Table S1. The phage library titer input and output numbers of each panning. The recovery rate ("a") was calculated as recovered phage divided by input phage.

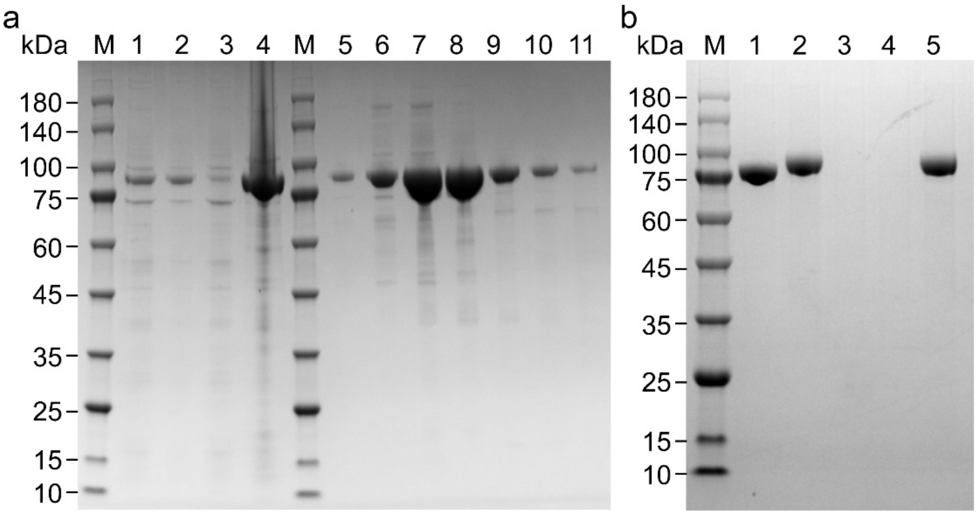

Figure S1. SDS-PAGE analysis of TfR1 purification and biotinylation. The TfR1 protein used as the antigen for phage display screening was expressed, purified, and subsequently biotinylated in our laboratory. The expressed protein corresponds to the extracellular domain of human TfR1, derived from UniProt entry P02786 (TFR1_HUMAN, residues 89 – 760 of TfR1). To obtain the target protein required for the experiments, the existing TfR1 recombinant expression plasmid in our laboratory, encoding the His_6-tagged extracellular domain of TfR1, was transfected into HEK 293F cells cultured in 100 mL of growth medium using PEI. After 5 days of culture, the cell culture supernatant was collected, and the protein was sequentially purified by Ni-NTA affinity chromatography and size-exclusion chromatography, followed by concentration using ultrafiltration. The purified TfR1 recombinant protein was labeled using N-hydroxysuccinimide biotin (NHS-biotin), and unreacted free NHS-biotin was subsequently removed using a PD-10 desalting column. To verify successful biotinylation of the TfR1 recombinant protein, a pull-down assay was performed using streptavidin magnetic beads. (a) Purification of recombinant TfR1 protein. Lane M: protein marker; Lanes 1 – 4: Ni-NTA affinity-purified samples; Lanes 5 – 11: samples further purified by size-exclusion chromatography using an SH-100 column. (b) Pull-down assay of biotinylated TfR1 protein. Lane M: protein marker; Lane 1: purified TfR1 protein; Lane 2: biotinylated TfR1 protein; Lane 3: supernatant after incubation with streptavidin magnetic beads; Lane 4: wash fraction from streptavidin magnetic beads; Lane 5: proteins eluted from streptavidin magnetic beads after heating.

a

Heavy chain of T8IgG1

MGWSCIILFLVATATGVHSEVQLLESGGGLVQPGGSLRLSCAASGFTFSXXXXXWVRQAPGKGLE
WVSXXXXXXXXXXYADSVKGRFTISRDNSKNTLYLQMNSLRAEDTAVYYCARXXXXXXXXFDYW
GQGTLVTVSSASTKGPSVFPLAPSSKSTSGGTAALGCLVKDYFPEPVTVSWNSGALTSGVHTFPA
VLQSSGLYSLSSVVTVPSSSLGTQTYICNVNHKPSNTKVDKKVEPKSCDKTHTCPPCPAPELLGG
PSVFLFPPKPKDTLMISRTPEVTCVVVDVSHEDPEVKFNWYVDGVEVHNAKTKPREEQYNSTYR
VVSVLTVLHQDWLNGKEYKCKVSNKALPAPIEKTISKAKGQPREPQVYTLPPSRDELTKNQVSLTC
LVKGFYPSDIAVEWESNGQPENNYKTTPPVLDSDGSFFLYSKLTVDKSRWQQGNVFSCSVMHEA
LHNHYTQKSLSLSPGK

b

light chain of T8IgG1

MGWSCIILFLVATATGVHSEIVLTQSPGTLSLSPGERATLSCRASXXXXXXXLAWYQQKPGQAPKL
LIYXXXXRATGIPDRFSGSGSGTDFTLTISRLEPEDFAVYYCQQXXXXXXTFGQGTKVEIKRTVAAP
SVFIFPPSDEQLKSGTASVVCLLNNFYPREAKVQWKVDNALQSGNSQESVTEQDSKDSTYSLSS
TLTLSKADYEKHKVYACEVTHQGLSSPVTKSFNRGEC

Figure S2. Amino acid sequences of T8IgG1 library. The signal peptide and complementarity-determining regions (CDRs) are underlined. X represents the randomized amino acid positions in the generated scFv phage library. (a) Heavy-chain amino acid sequence of T8IgG1. (b) Light-chain amino acid sequence of T8IgG1.

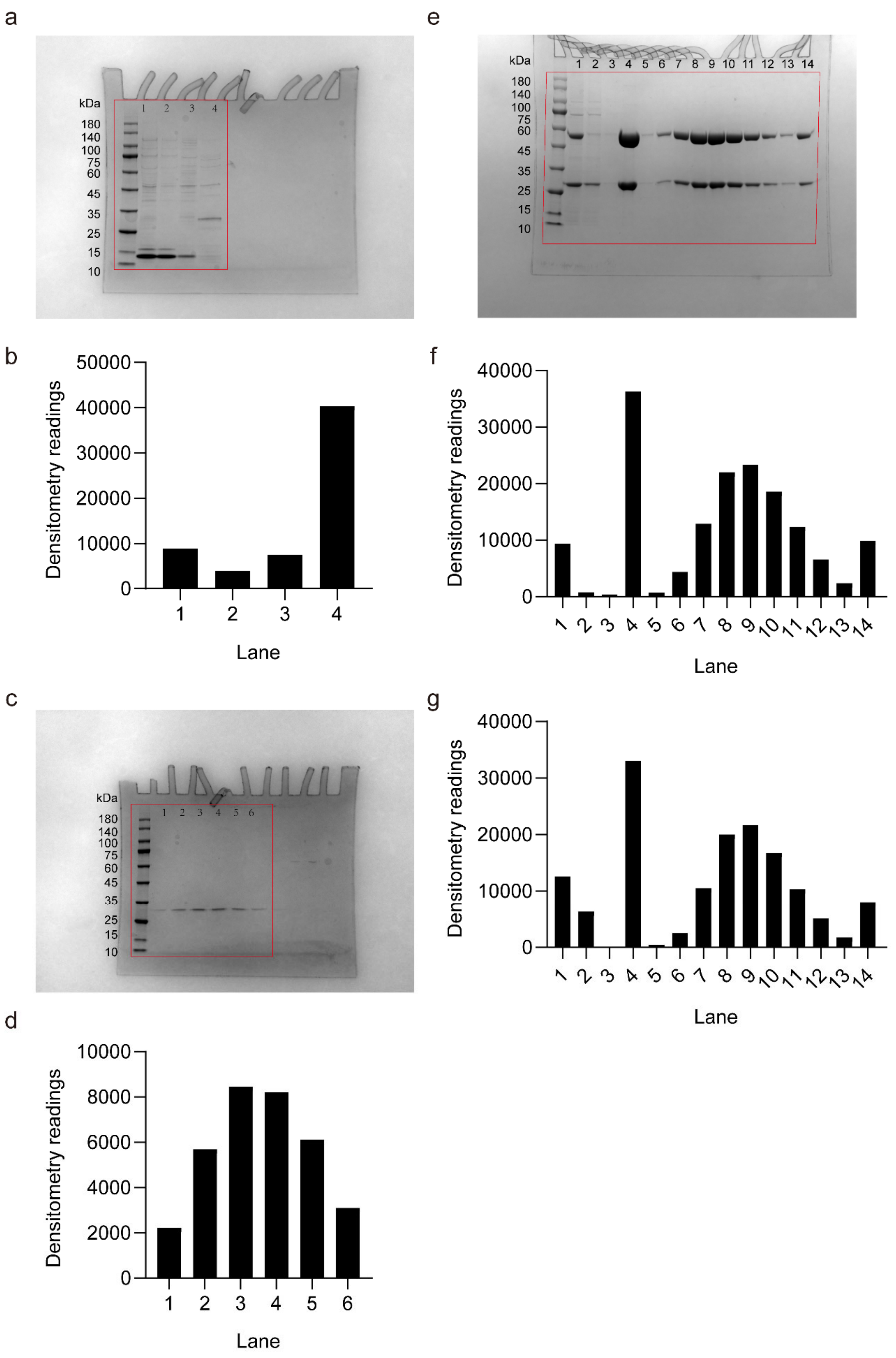

Figure S3. The original SDS-PAGE images of T8scFv and T8IgG1, along with the analysis results of the densitometry readings of the target bands. (a) The original image of Figure 3a in the main text. (b) Densitometry readings of the T8scFv (27 kDa) band in Supplementary Figure

3a (Lane1-4). (c) The original image of Figure 3b in the main text. (d) Densitometry readings of the T8scFv (27 kDa) band in Supplementary Figure 3c (Lane1-6). (e) The original image of Figure 3c in the main text. (f) Densitometry readings of the T8IgG1 heavy chain (50 kDa) band in Supplementary Figure 3e (Lane1-14). (g) Densitometry readings of the T8IgG1 light chain (25 kDa) band in Supplementary Figure 3e (Lane1-14).

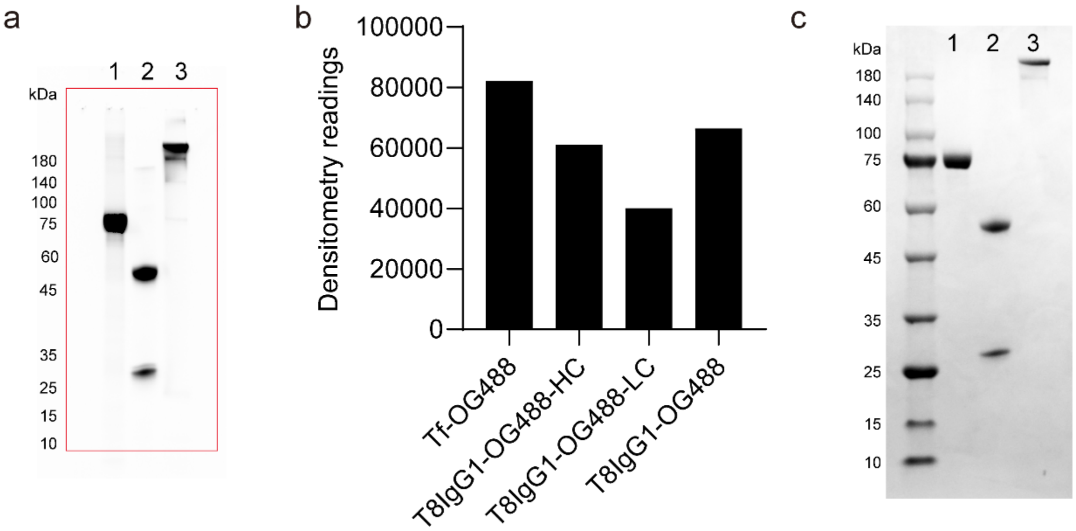

Figure S4. Fluorescent scanning of the original SDS-PAGE of Tf-OG488 and T8IgG1-OG488, along with the analysis results of the densitometry readings of the target bands. (a) The original image of Figure 4a in the main text. (b) Densitometry readings of the Tf-OG488 band, the T8IgG1-OG488 heavy and light chain (HC and LC) bands, and the T8IgG1-OG488 full antibody band in Supplementary Figure 4a. This native image was pseudocolored according to the scanning wavelength and presented in the main text. (c) Coomassie staining of the gel, shown in Supplementary Figure 4a, to visualize non-fluorescent protein markers.

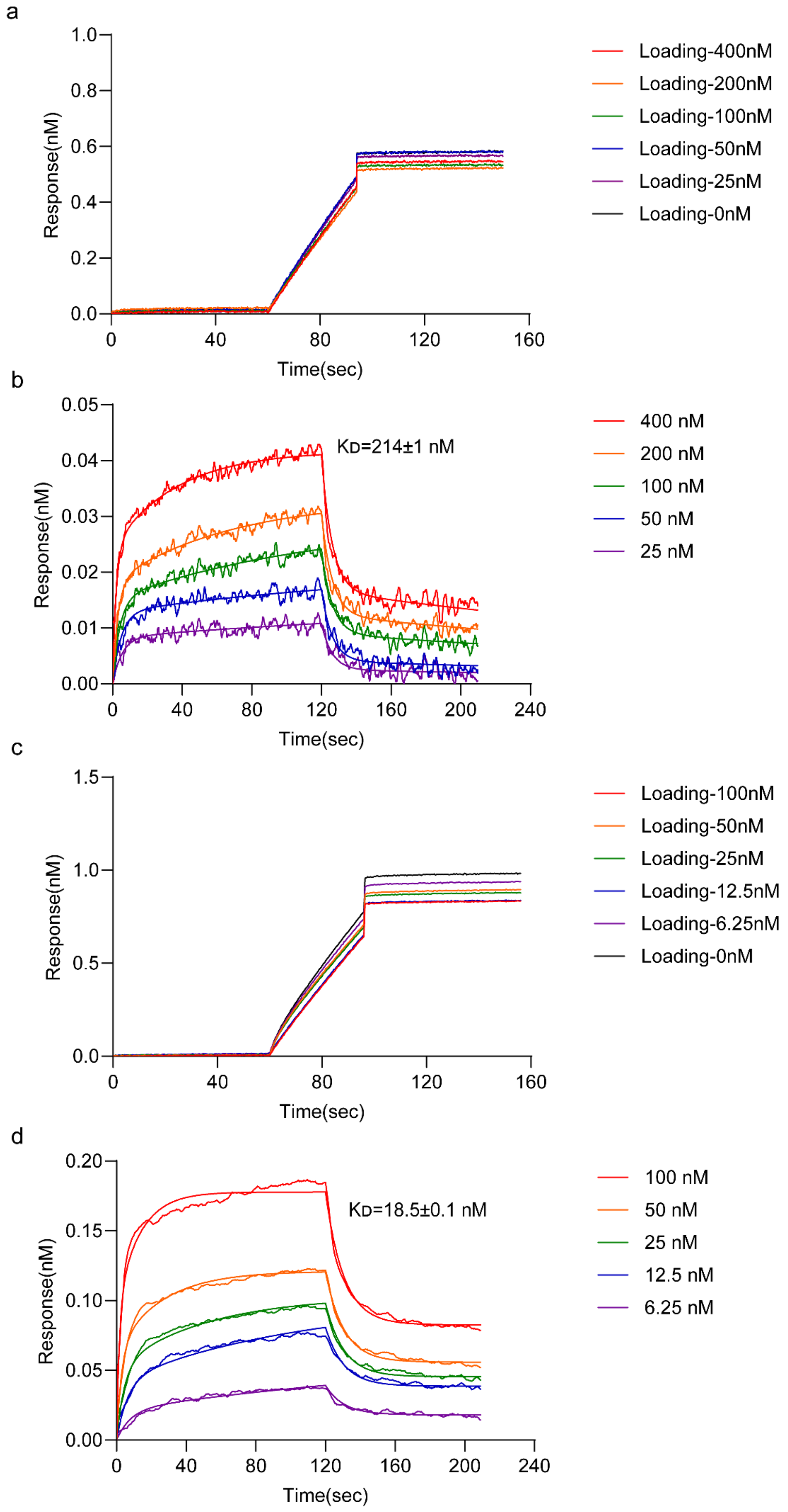

a
Response(nM)
Time(sec)
Loading-400nM
Loading-200nM
Loading-100nM
Loading-50nM
Loading-25nM
Loading-0nM
b
KD=214±1 nM
400 nM
200 nM
100 nM
50 nM
25 nM
c
Loading-100nM
Loading-50nM
Loading-25nM
Loading-12.5nM
Loading-6.25nM
Loading-0nM
d
KD=18.5±0.1 nM
100 nM
50 nM
25 nM
12.5 nM
6.25 nM

Figure S5. Immobilization, association–dissociation, and corresponding fitted kinetic curves of T8scFv and T8IgG1 in Octet experiments. (a) Immobilization curve of T8scFv. (b) Association–dissociation, and corresponding fitted kinetic curves of T8scFv. (c) Immobilization curve of T8IgG1. (d) Association–dissociation, and corresponding fitted kinetic curves of T8IgG1.

**Name**	**K_D(nM)**	**K_{on}(1/Ms)**	**K_{off}(1/s)**	**X^2**	**R^2**
T8scFv	214±1	771300	0.1652	0.0012	0.9905
T8IgG1	18.5±0.1	761100	0.0959	0.0059	0.9951

Table S2. Kinetic parameters of T8scFv and T8IgG1 determined by Octet analysis. K_{on} represents the association rate constant, Koff the dissociation rate constant, and K_D the equilibrium dissociation constant (K_{off}/K_{on}), reflecting the binding affinity. X^2 denotes the fitting residual, and R^2 indicates the goodness of fit between the model and experimental data.

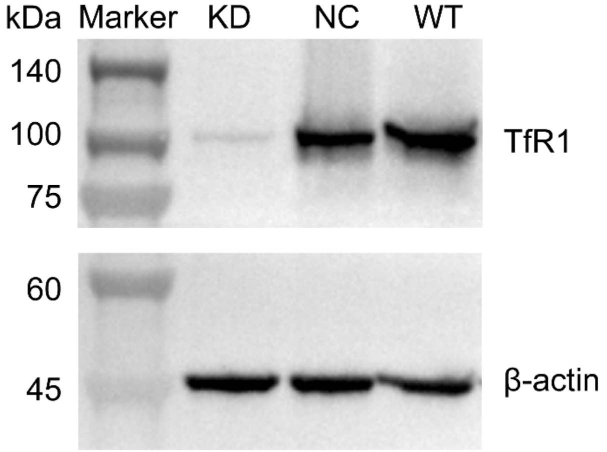

Figure S6. Western blot analysis of TfR1 expression suppression by TfR1-targeting siRNA in K562 cells. The KD group represents K562 cells transfected with TfR1-targeting siRNA, the NC group represents K562 cells transfected with a non-specific control sequence (NC siRNA), and the WT group represents untreated K562 cells.

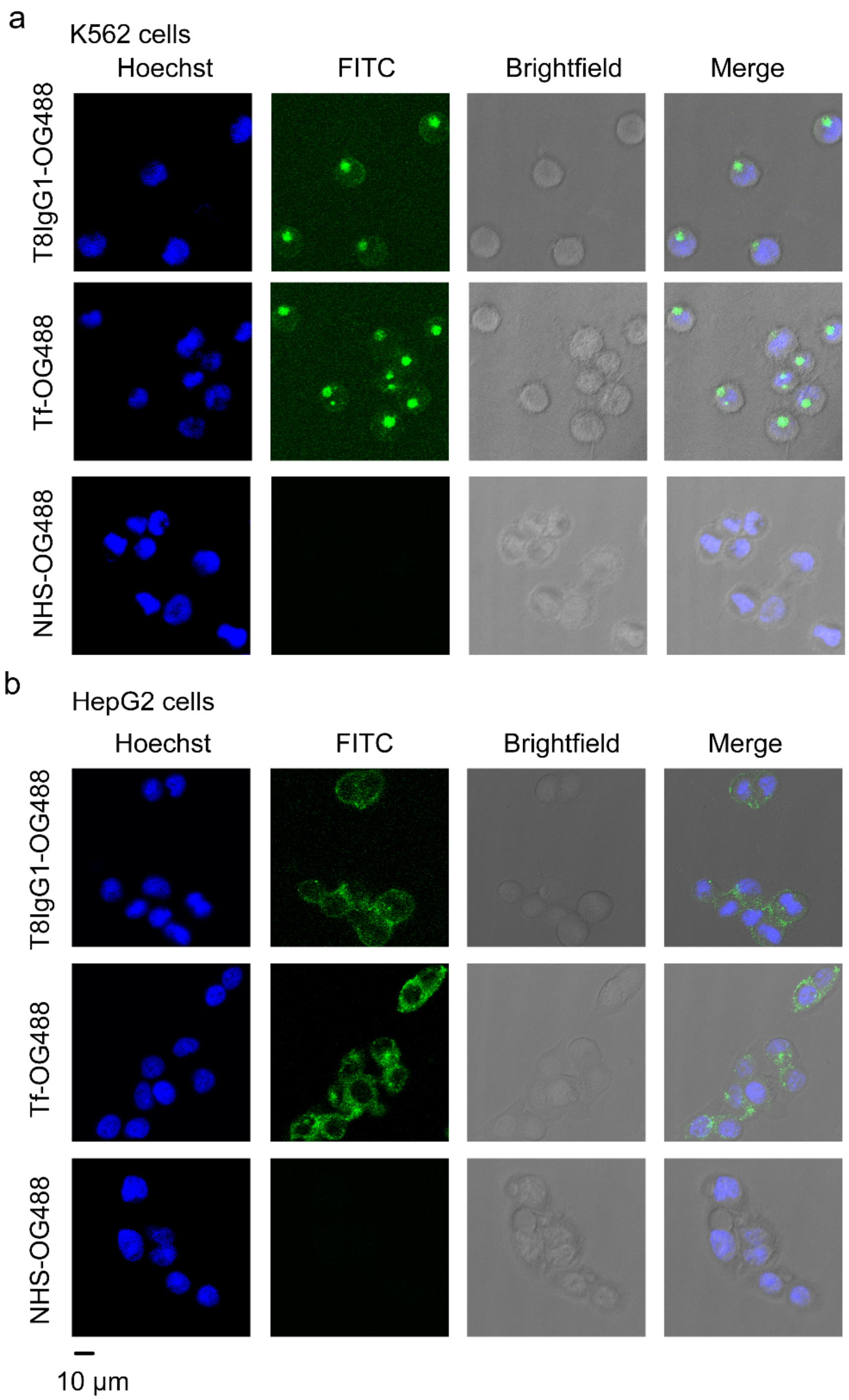

Figure S7. Evaluation of antibody internalization with bright-field images. T8IgG1-OG488 was incubated with K562 (a) and HepG2 (b) cells for 2 hours, followed by PBS washing and cell fixation. Nuclei were stained with Hoechst, and FITC signal distribution was observed under a fluorescence microscope. Green fluorescence indicates the location of T8IgG1-OG488, while blue fluorescence marks the nuclei. The green fluorescence is predomi-nantly distributed in the cytoplasmic region, indicating strong cellular internalization capability of the antibody. In the positive control group with Tf-OG488, green fluorescence is also observed in the cytoplasm. In the negative control group treated with NHS-OG488 alone, no significant green signal is

detected, further confirming that the internalization capability is an intrinsic property of T8IgG1.

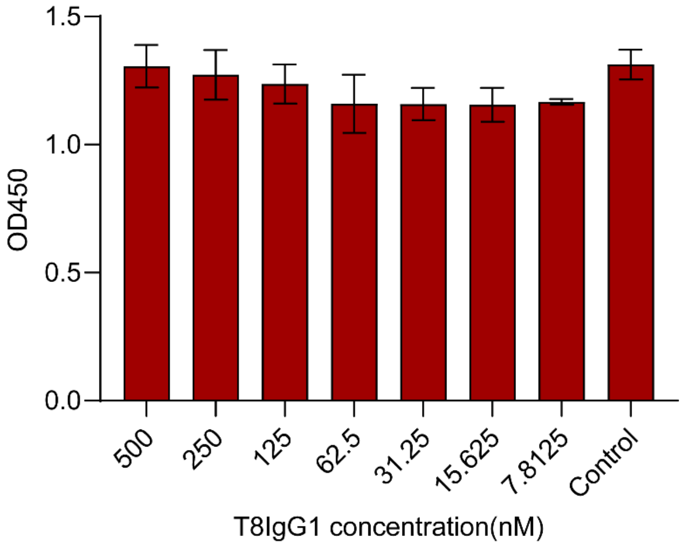

Figure S8. Competitive ELISA assessment of the potential interference of T8IgG1 with Tf–TfR1 binding. Tf (0.5 μg/well) was immobilized on ELISA plates at 4 °C overnight and subsequently blocked. Biotinylated TfR1 (100 nM) was pre-incubated with varying concentrations of T8IgG1 at 37 °C for 1 h, and the mixtures were then added to the Tf-coated wells for an additional 1 h. Wells incubated with biotinylated TfR1 (100 nM) alone served as the control. After washing, bound biotinylated TfR1 was detected using streptavidin-HRP (1:5000), followed by TMB development and termination with concentrated H_2SO_4. Absorbance was measured at 450 nm. Across the tested concentration range, no significant differences in OD_{450} values were observed between T8IgG1-treated wells and control wells.
